# Evaluation of the soil microbiome of three raised beaches in the Devon Island Lowland, High Arctic, Canada

**DOI:** 10.1371/journal.pone.0336235

**Published:** 2025-11-05

**Authors:** Laura Maretto, Saptarathi Deb, Andrea Squartini, Giuseppe Concheri, Piergiorgio Stevanato, Serenella Nardi, Stefania Cocco, Giuseppe Corti

**Affiliations:** 1 Department of Agronomy, Food, Natural Resources, Animals and Environment – DAFNAE, University of Padua, Padua, Italy; 2 Department of Agricultural, Food and Environmental Sciences, Polytechnic University of Marche, Ancona, Italy; 3 Agriculture and Environment Centre, Council for Agricultural Research and Economics – CREA, Rome, Italy; Maria Curie-Sklodowska University: Uniwersytet Marii Curie-Sklodowskiej, POLAND

## Abstract

The Arctic region is characterized by severe temperatures and a unique ecosystem with largely unexplored microbiomes. Whereas soil microbiomes in temperate regions play key roles in nutrient cycling, organic matter decomposition, greenhouse gas fluxes, and overall ecosystem functioning, Arctic microbiomes remain poorly understood, highlighting the need for a thorough characterization to better predict and manage soil health and resilience. In this study, we compared the microbial profiles of three raised beaches on Devon Island (Nunavut, Canadian Arctic Archipelago), which emerged sequentially between eight and two thousand years ago, to assess their similarities and differences. Samples were collected by genetic horizons along excavations from the top layer to the permafrost. For each horizon, total soil DNA, 16S gene copies dPCR quantification, 16S metabarcoding, and functional prediction were carried out. Total DNA quantification revealed a consistently comparable concentration of genetic material across the three soil beaches (AB2 μ = 2.28 ± 5.44 μg ∙ g^-1^, AB1 μ = 4.71 ± 2.35 μg ∙ g^-1^, AB3 μ = 5.44 ± 2.91 μg ∙ g^-1^), regardless of site age (AB2 = 2,360 YBP, AB1 = 6,726 YBP, AB3 = 8,410 YBP). Conversely, clear differences emerged by comparing the different horizons at each site. The hierarchical cluster analysis based on the Bray-Curtis dissimilarity matrix revealed a clear separation between surface and deep horizons. The core microbiome analysis highlighted Actinobacteria, Proteobacteria, and Firmicutes as the three predominant phyla accounting for relative abundances of 42%, 22%, and 18%, respectively. Remarkable evidence was the unexpectedly high taxonomic diversity that was recorded in these sites and that surprisingly matched with the commonly observed values in soils of temperate regions. Since these stony shores developed under cold, life-limiting conditions, their apparent microbial richness raises doubts about the potential biases in inferring physiological contexts and active biodiversity directly inferred from culture-independent DNA-based studies. The reason is that such inventories can be possibly inflated, in all environments, by chronically accumulated cells from passive immigration events through atmospheric discharge.

## Introduction

The Arctic region is a delicate ecosystem characterised by low temperatures, limited biodiversity, and extensive ice and snow cover [1–3]. The persistence of a permafrost layer in the High Arctic terrain exposes the active layer to intense frost-driven processes, including cryoturbation. As a result, the soil typically exhibits circumvoluted and uneven horizons, material injection, stone upheaval, and soil blending. Nonetheless, some areas contain non-cryoturbated soils, usually characterized by a vertical arrangement of the active layer made of A–B–C master horizons. Such soils are commonly found in coastal regions [4]. Because of its severe conditions, this area sustains only a restricted array of plant species, which is mainly composed of mosses and lichens.

Currently, the Arctic is undergoing rapid transformations because of climate change. Since 1979, Arctic temperatures have increased at roughly four times the global average [5], leading to a progressive deepening of the permafrost layer [6,7]. This change has extensive consequences that affect biological communities, biogeochemical processes, and the overall ecosystem functioning [8]. For instance, raising in air and water temperatures have altered the migration patterns of predators and preys, leading to shifts in community composition as Arctic species are gradually supplanted by southern counterparts [9]. Permafrost thawing also raised environmental risks [10].

Several studies have examined soil bacterial communities and their role in nutrient cycling [11–13] and soil fertility [14,15], and their ecosystem functioning has been widely documented by the scientific community [16–18]. As with non-polar microbial communities, the Arctic soils’ microbiome, despite the low activity rates due to environmental conditions, is responsible for organic matter decomposition [19], and essential nutrient cycling [20,21]. Consequently, even minor alterations induced by global warming, such as augmented water availability and active layer depth, might cause an increase in the methanogenic bacteria abundance in soils, resulting in a boosted CH_4_ production [22], which has about 30-fold higher global warming potential (GWP) when compared to CO_2_ [23]. Thus, a better understanding of the Arctic soil microbial communities is key to predict changes in soil health and resilience across a wide range of latitudes.

This work aimed to investigating the soil microbial communities in the horizons forming both active layer and permafrost along a chronosequence represented by three raised beaches dated between 2,360 and 8,410 years before present (YBP). Soil at the raised beaches did not show signs of cryoturbation and displayed a surface with desert pavement with stones coloured by desert varnish. This study employed a multidisciplinary approach, encompassing soil physicochemical analyses and molecular biology techniques such as total DNA assessment and 16S gene copies quantification, 16S multi-amplicon metabarcoding, and environmentally relevant functions prediction.

## Materials and methods

### Study area

**Fig 1:**
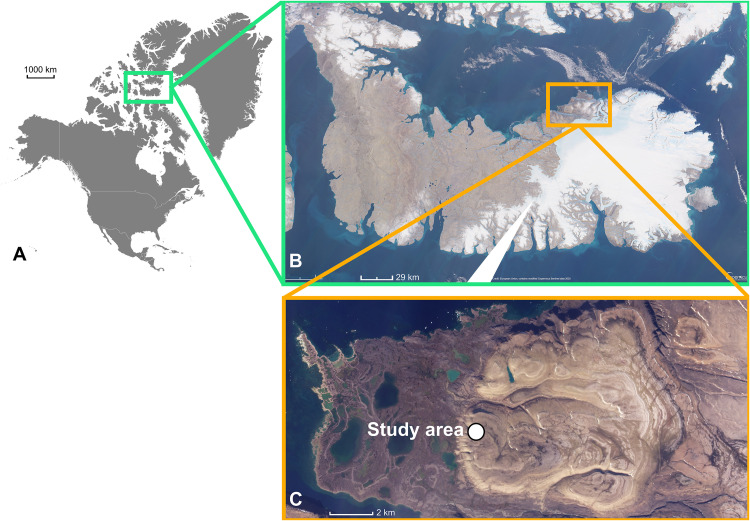
Aerial photograph of the study area located on Devon Island in the Canadian High Arctic region of Nunavut. **(A)** Map of North America with the indication of the experimental zone (Vemaps © https://vemaps.com/north-america-continent/na-c-04); **(B)** Devon Island photographed by Sentinel 2 (Copernicus project); **(C)** Detailed photograph of the studied area, Image © 2024 Planet Labs PBC.

The study was conducted on three contiguous raised beaches (identified as AB1, AB2, and AB3) situated in the area named “seagull beach”, which is part of the lowland of Devon Island, in the Canadian region of Nunavut (Fig 1). The research and accommodation at Truelove Camp were authorized by the Arctic Institute of North America, within the framework of the project OPP-93–00045 funded by the national Science Foundation.

Devon Island, part of the Queen Elizabeth Islands located in the High Arctic, has a surface of 56,000 km^2^. It features a prevailing continental climatic condition shaped by the surrounding sea, which remains frozen for more than ten months per year, and by the influence of a cold circumpolar vortex that blows from west to east [24]. Although the coastal and lower-lying regions display biological and soil conditions resembling the wet tundra [25], the rest of the island is characterized by an ice cap and a barren plateau. Climatic records of Truelove, one of the coastal lowlands on Devon Island, indicate an annual mean air temperature of approximately −16.0°C, and an annual mean precipitation of 185 mm [24,26]. Geology of the island comprises Cambrian and Ordovician sedimentary rocks attributed to the Cass Fjord Formation [4]. A glacial ice cap, which is still present in the northeast of the island, covered Devon Island. Approximatively 9,000 years ago, the deglaciation process began, transforming the glacial deposits and the underlying bedrock into permafrost [27]. Concurrently, the ice-cap retreat induced a glacio-isostatic crustal rebound phenomenon [28] that progressively originated raised beaches in the coastal area. Nowadays the raised beaches lie at elevations between 1–2 and 50 meters above sea level, reflecting their emergence time, host permafrost, and are made of 80–90% rock fragments (particles>2 mm). Often, these beaches contain sub-rounded pebbles and blocks. The fine earth fraction is limited, representing only about 5–10% of the beach soil overall composition. This fine earth is typically found within the silt/sand caps formed through illuviation on the surface of sub-superficial pebbles and blocks. Due to the skeletal nature of these soils, they exhibit exceptional drainage characteristics, preventing the active layer from experiencing waterlogging periods. This favourable drainage condition contributes to the absence of cryoturbation phenomena able to mix soil horizons. As a result, the soils of the raised beaches preserve a vertical A-B-C master horizons sequence formed through non-cryoturbated pedogenesis that operated on the glacial till exposed after the ice retreat and isostatic rebound. Then, it is also presumable that all materials within the permafrost have remained unaltered since deglaciation, while the active layer may have undergone translocations of organics and minerals due to processes like illuviation, root growth, and faunal activities such as burrowing.

The AB2 beach has an elevation of 3.22 meters, was radiocarbon dated to 2,360 YBP [29], showed the ice table at 60–63 cm of depth, and was characterized by a surface covered for 50–60% by crusty lichens, and for 7–10% by vascular plants such as *Cerastium* spp., *Saxifraga* spp., and *Salix* spp., while the rest of the surface was barren with a poorly developed desert pavement. The AB1 beach is at an elevation of 22.44 meters, dated back 6,726 YBP [29], displayed the ice table at about 70 cm of depth, and was characterized by a surface covered by crusty lichens for 30–40% and by vascular plants such as *Dryas* spp., *Saxifraga* spp., and *Salix* spp. for 7–10%; the rest of the surface was barren with a rather well-developed desert pavement. The AB3 beach raises for 47.52 meters above sea level, was dated 8,410 YBP [29], showed the ice table at 79–80 cm of depth and displayed a surface cover made of crusty lichens for 10–20% and by vascular plants (*Dryas* spp., *Saxifraga* spp., and *Salix* spp.) for 7–10%; most of the AB3 surface (70–80%) was barren and presented a well-developed desert pavement. All the soils were classified as Typic Haplorthel [30].

### Field operations and sample collection

The study site selection was based on six excavations performed within each raised beach, covering an area of approximately 500 m^2^. At the designated location, a comprehensive identification of the surface vegetation was conducted, and two distinct soil trenches were excavated in each raised beach until the frost table was reached. After sampling the active layer, the trenches were kept open for three weeks, during which a camping gas lantern was used to thaw the permafrost. This step was essential since scrutiny and collection of the near-surface permafrost were key aspect of the study.

Soils from the three investigated sites were morphologically described according to the guidelines of Schoeneberger et al. [30], using the norms to codify the horizons defined by Soil Survey Staff in 2014 [31] and their main features are reported in S1 Table. A, B, C and E are the symbols for the master genetic horizons, BC is a transitional horizon. Other additional symbols refer to specific conditions of the master and transitional horizons; in particular, the “f” suffix stands for permanently frozen horizon – the permafrost. The extent of silt cap development was evaluated and categorized using a ranking system based on levels ranging from 1 to 5, relative to a height of the silt caps of 1–2, 3–4, 5–6, 7–8, and >8 mm thick. Carbonates pendants and stains were assessed by testing with 3 M HCl solution. Red, reddish, and purplish stains presence was quantified using the Munsell Soil Color Charts [32]. Approximately 3 kg of soil was collected from each horizon for a total of 40 samples gathered (AB2 soil: 2 profiles x 5 horizons; AB1 soil: 2 profiles x 8 horizons; AB3 soil: 2 profiles x 7 horizons). During the sampling activity, a pebble count was conducted on approximately 110 washed pebbles per horizon (S2 Table). Fieldwork also included volumetric horizon sampling to determine, in the laboratory, the bulk density of both fine-earth and skeletal fractions [33]. For each sample, after sieving at 2 mm the volume-based sample, the bulk density of the skeleton was determined by measuring its bulk volume by water displacement after sub-samples were completely water-saturated and, then, its weight at 105°C. The bulk density of the fine-earth fraction was calculated from the volume and weight difference between the total collected sample and the skeletal fraction.

### Soil physicochemical analyses

Soil samples were air-dried and sieved at a mesh size of 2 mm to separate the fine-earth from the rock fragments. To assess particle-size distribution, coarse, medium, and fine sands were separated through sequential sieving at mesh sizes of 0.5 mm, 0.25 mm, and 0.05 mm, respectively. Clay was separated from the silt by sedimentation in a suspension adjusted to a pH range of 8.8 to 8.9 using NaOH and maintained at 20°C. Soil pH was determined potentiometrically on water suspensions with a solid-liquid ratio of 1:2.5. The organic carbon (OC) content was determined using the Walkley–Black method. The determination of total Kjeldahl nitrogen (TKN) and micaceous nitrogen (MN) employed a modified approach described by Corti et al. [34]. The available phosphorus (P) quantification was carried out following the Olsen method [35].

### Total soil DNA extraction, 16S gene copies quantification, 16S metabarcoding, and function prediction

The extraction of total soil DNA was performed using the DNeasy PowerSoil Pro Kit (Qiagen GmbH, Hilden, Germany, DE), following the manufacturer’s guidelines. Extracted and purified nucleic acids were quantified with a Qubit Flex fluorometer (Thermo Fisher Scientific, Carlsbad, CA) paired with the Qubit 1x dsDNA High Sensitivity Assay Kit (Thermo Fisher Scientific, Carlsbad, CA).

The abundance of the 16S gene was quantified by digital PCR (dPCR) using a QIAcuity One, 5plex Device paired with QIAcuity Nanoplate 26k and QIAcuity EvaGreen (EG) PCR Kit (Qiagen GmbH, Hilden, Germany, DE). The universal primer assay proposed by Johnson et al. [36] was used for 16S gene copies quantification.

16S rDNA multi-amplicon metabarcoding libraries were prepared using the 16S Ion Metagenomics Kit (Thermo Fisher Scientific, Carlsbad, CA) and sequenced on the Ion GeneStudio S5 System (Thermo Fisher Scientific, Carlsbad, CA) employing an Ion 520 chip.

Raw reads processing followed the pipeline outlined by Maretto et al. [37]. The uBAM files sourced from the Ion GeneStudio platform were converted into FASTQ format using the samtools bamtofastq (v1.10) by Li et al. [38]. Sequencing primers were removed by trimming 20 nucleotides from both ends of the raw reads using cutadapt (v3.5) [39]. The “Quantitative Insights Into Microbial Ecology 2” (QIIME2) (v2020.08) [40] pipeline was subsequently used to analyse the trimmed raw reads. Imported reads were denoised and dereplicated using the “qiime dada2” plugin followed by taxonomic classification of Amplicon Sequence Variants (ASVs) by a “classify-consensus-blast” plugin using SILVA SSU (version 138.1) [41] as the reference database. Due to a low number of reads within each sample, the two AB2-BC1 biological replicates were merged using the “qiime feature-table group” plugin. Afterwards, the resulting feature abundance and taxonomic assignment tables were exported and analysed using RStudio (version R-4.2.2) [42,43] along with *tibble* [44] and *TaxaPhyloseq* [45] R-packages. Read count normalization was performed with the *DESeq2* R-package [46], while *MicrobiotaProcess* package [47] was used to calculate diversity indices and perform beta-diversity analyses. All graphical visualizations were generated using the *ggplot2* R-package [48]. In this study, we use the term ASV to denote the deepest achievable resolution level of observed phylotypes. The collective term ‘taxa’ is used in a broader sense, encompassing entities that may fall above or beyond the species rank. The issues linked to the potential reads redundancy from given organisms, arising from multiple ribosomal copies or to the use of a multi-amplicon approach, should be considered when comparing results obtained by different sequencing strategies and bioinformatics pipelines.

Functional prediction analysis was performed using FAPROTAX online database (version 1.2.7) [49] to estimate the number of ecologically relevant functions within each sample.

### Statistical analyses

Statistical analyses were conducted using RStudio and the *dplyr* package [50]. Spearman’s rank correlation coefficient [51] was used to assess ranking correlations among the biological parameters. Significant differences in mean values among site clusters (AB2, AB1, AB3) and horizon clusters (A, Bw, BC, BCf) were tested using the non-parametric Kruskal-Wallis test [52].

## Results

### Soil morphology

General information and morphology of the soils at the raised beaches are provided in S1 [53] and S2 Tables. The three beaches differ by age of emersion and are all located within a 700-meters distance. Soils showed a well-developed sequence of A-B-BC horizons, with no evidence of cryoturbation. The frozen soil (BCf horizons) occurred at depths of 60–63 cm in the youngest beach, 68–70 cm in the intermediate one, and 79–80 cm in the oldest. As no evidence of annual ice was observed (*sensu* [54]), the frozen horizons were interpreted as permafrost, and the soil were therefore classified as Gelisols [30]. The soils consisted predominantly of skeletal particles, containing less than 10% fine earth (mainly concentrated in the silt caps), and diffuse large voids (open work). A weak to very weak the fine-earth structure was restricted to the superficial horizons. Roots were distributed throughout the soil to depyhs of 45–60. Pendants and yellowish-brown staining occurred in nearly all horizons except in the frozen ones. Pebble counts indicated that horizons were dominated by crystalline and dolomitic rock fragments, roughly more dolomitic than crystalline in the AB2, and more crystalline in the AB1 and AB3. Other fragments’ rock types such as sandstone, diabase, breccia, and shales were less abundant, although in some horizons their proportion reached 20% or more (S2 Table).

### Soil physicochemical comparative analyses

**Fig 2:**
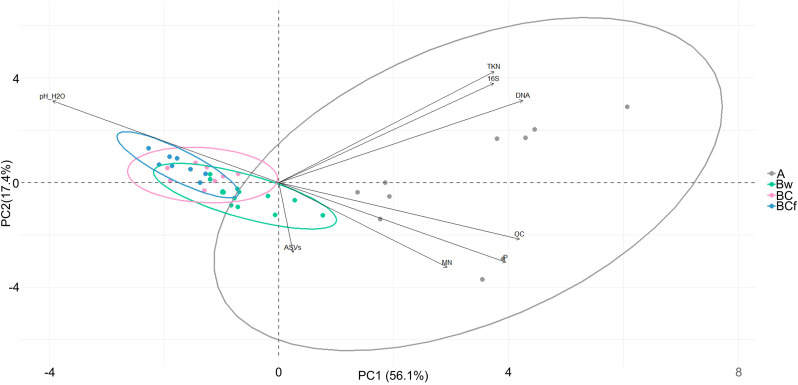
Principal Component Analysis (PCA) biplot to evaluate differences among the identified horizons. Biplot illustrating the spatial clustering of the analysed samples and their variables based on their chemical and biological properties and labelled according to the profile horizons: A horizon in grey, Bw horizon in green, BC horizon in pink, BCf horizon in blue. Samples located on the same side of a particular variable exhibit higher values for that variable.

Soil physicochemical properties are reported in S3 and S4 Tables, and differences among sites and horizons are summarised in Table 1. Soil texture was dominated by sand, which represented more than 90% of the fine earth fraction; with silt comprising the remainder and clay being virtually absent. The comparative analysis showed that the site-based (horizontal dimension) clustering among the three beaches revealed no significant differences, except for MN, which represents the isomorphic NH_4_-substitution of part of the K ions in mica interlayers [34]. AB3 beach exhibited a significantly higher (H = 21.76, *p* < 0.001) MN content (9.4 ± 0.7 mg·kg^-1^) than AB1 and AB2 beaches (4.9 ± 0.7 and 4.1 ± 0.4 mg·kg^-1^, respectively). In contrast, the horizon-based (vertical dimension) clustering (Fig 2) revealed several significant differences among the investigated parameters. pH increased significantly (H = 28.79, *p* < 0.001) from the A horizon (pH 7.61 ± 0.06) to the BC and BCf horizons (pH 8.26 ± 0.04 and 8.35 ± 0.05, respectively). Conversely, OC, available P, and MN contents showed an opposite trend compared to the pH. OC content decreased (H = 33.14, *p* < 0.001) from 8.23 ± 1.46% in the A horizon to 0.08 ± 0.08 and 0.07 ± 0.01% in the BC and BCf horizons, respectively. Available P decreased significantly (H = 31.65, *p* < 0.001) from 7.1 ± 0.6 mg·kg^-1^ in the A horizon to 0.4 ± 0.2 mg·kg^-1^ in the BCf horizon. MN content decreased (H = 7.75, *p* ≤ 0.05) from 8.8 ± 1.0 mg·kg^-1^ in the A horizon to 4.6 ± 1.2 and 4.4 ± 1.2 mg·kg^-1^ in the BC and BCf horizons. TKN content was significantly enriched (H = 9.04, *p* < 0.05) in the A horizon (1.73 ± 0.51 g·kg^-1^) compared to Bw (0.23 ± 0.08 g·kg^-1^), BC (0.33 ± 0.11 g·kg^-1^), and BCf (0.16 ± 0.03 g·kg^-1^) horizons.

**Table 1:** Physico-chemical comparative analyses (by site and by depth) performed on the soil samples from the raised beaches at “seagull beach” area, Devon Island Truelove Lowland, High Arctic Canada.

Site	Skeleton	Fine Earth Fraction %					pH_(H2O)_		Organic C		Kjeldahl N		Micaceous N***		Available P	
	%	Coarse Sand	Medium Sand	Fine Sand	Silt	Clay			%		g·kg^-1^		mg·kg^-1^		mg·kg^-1^	
AB1	66.0 ± 4.5	82.3 ± 1.3	6.6 ± 0.40	8.5 ± 0.85	2.6 ± 0.5	<0.1	8.08 ± 0.07	a	1.55 ± 0.61	a	0.67 ± 0.25	a	4.9 ± 0.7	b	4.4 ± 0.7	a
AB2	81.0 ± 1.5	69.3 ± 4.2	6.8 ± 0.83	16.9 ± 2.99	7.0 ± 1.0	<0.1	8.02 ± 0.08	a	1.38 ± 0.71	a	0.26 ± 0.05	a	4.1 ± 0.4	b	2.5 ± 0.4	a
AB3	72.0 ± 4.1	81.9 ± 1.9	8.2 ± 0.69	6.0 ± 0.78	4.5 ± 0.9	<0.1	8.06 ± 0.11	a	4.13 ± 1.59	a	0.80 ± 0.37	a	9.4 ± 0.7	a	3.6 ± 0.9	a
Horizon	Skeleton	Fine Earth Fraction %					pH_(H2O)_***		Organic C***		Kjeldahl N**		Micaceous N*		Available P***	
	%	Coarse Sand	Medium Sand	Fine Sand	Silt	Clay			%		g·kg^-1^		mg·kg^-1^		mg·kg^-1^	
A	66.7 ± 6.2	75.0 ± 3.0	8.1 ± 0.6	13.5 ± 2.5	4.3 ± 0.9	<0.1	7.61 ± 0.06	c	8.23 ± 1.46	a	1.73 ± 0.51	a	8.8 ± 1.0	a	7.1 ± 0.6	a
Bw	69.2 ± 4.0	74.7 ± 2.3	7.4 ± 0.4	12.0 ± 1.6	5.8 ± 1.0	<0.1	8.10 ± 0.04	b	0.93 ± 0.20	b	0.23 ± 0.08	b	6.5 ± 0.6	ab	4.1 ± 0.4	b
BC	70.5 ± 3.7	81.1 ± 3.5	7.8 ± 0.6	7.5 ± 1.7	3.7 ± 1.2	<0.1	8.26 ± 0.04	a	0.08 ± 0.08	c	0.33 ± 0.11	b	4.6 ± 1.2	b	2.00 ± 0.3	c
F	84.2 ± 3.5	88.9 ± 2.1	5.2 ± 1.3	3.3 ± 0.7	2.5 ± 0.3	<0.1	8.35 ± 0.05	a	0.07 ± 0.01	c	0.16 ± 0.03	b	4.4 ± 1.2	b	0.4 ± 0.2	d

Comparisons for statistical differences among the three beach sites (top) or among the four horizon types, irrespective of site (bottom) are shown. Means with the same letter in the vertical comparison are not significantly different at the Kruskal-Wallis test. Significance level: *p ≤ 0.05, **p < 0.01, ***p < 0.001.

### Total soil DNA extraction, 16S gene copies quantification, 16S metabarcoding, and function prediction

Total soil DNA content (Table 2) did not differ significantly among the three beaches. In contrast, the comparison among horizons revealed that in the A horizon there was a significant (H = 28.69, *p* < 0.001) 64-fold increase in total soil DNA when compared to the Bw horizon (17.0 ± 3.32 *vs*. 0.265 ± 0.149 µg·g^-1^), and the increase reached up to ~700-fold when the A horizon was compared to the BC and BCf horizons (0.024 ± 0.001 and 0.022 ± 0.001 µg·g^-1^, respectively). The number of 16S gene copies assessed by dPCR showed the same vertical trend as total soil DNA (H = 25.76, p < 0.001). No significant difference in the number of 16S copies were observed among sites. However, in the vertical dimension, the A horizon yielded a number of 16S copies (3.29 × 10^7^ ± 9.96 × 10^6^) that was 164 times higher than the Bw horizon (1.99 × 10^5^ ± 1.56 × 10^5^) and approximately 2500 times higher than the value observed in the BC and BCf horizons (1.31 × 10^4^ ± 2.05 × 10^3^ and 1.35 × 10^4^ ± 3.14 × 10^3^, respectively). DNA content and dPCR targeting the 16S gene copies represent the quantitative assessment. In contrast, the analysis of the 16S gene in the soil samples yielded 30,562,018 single-end reads with an average 239 nucleotide length. The number of amplicon sequence variants (ASVs) representing bacterial phylotype features, was not significantly different among the three beach soils. This finding is noteworthy given that their age is instead very different, spanning from 2,360–8,410 years. Only in the comparison, grouping the samples by horizons (A, Bw, BC, BCf), a significant difference (*p* < 0.001) between the two shallower horizons and the two deeper horizons arose (Table 2).

**Table 2:** Results of the biological analyses results performed on the soil samples from the raised beaches at “seagull beach” area, Devon Island Truelove Lowland, High Arctic Canada. Comparisons for statistical differences among the three beach sites (top) or among the four horizon types, irrespective of site (bottom) are shown.

Site	DNA Concentration		*16S* gene copies		ASVs	
	µg·g^-1^					
AB1	4.71 ± 2.35	a	8.59 × 10^6^ ± 4.57 × 10^6^	a	5,657 ± 982	a
AB2	2.28 ± 1.51	a	5.82 × 10^6^ ± 4.28 × 10^6^	a	4,767 ± 1,338	a
AB3	5.44 ± 2.91	a	9.72 × 10^6^ ± 7.51 × 10^6^	a	3,247 ± 714	a
Horizon	DNA Concentration***		*16S* gene copies***		ASVs***	
	µg·g^-1^					
A	17.0 ± 3.32	a	3.29 × 10^7^ ± 9.96 × 10^6^	a	6,639 ± 622	a
Bw	0.265 ± 0.149	b	1.99 × 10^5^ ± 1.56 × 10^5^	b	6,135 ± 1,187	a
BC	0.024 ± 0.001	c	1.31 × 10^4^ ± 2.05 × 10^3^	c	2,075 ± 389	b
BCf	0.022 ± 0.001	c	1.35 × 10^4^ ± 3.14 × 10^3^	c	1,508 ± 264	b

Means with the same letter in the vertical comparison are not significantly different at the Kruskal-Wallis test. Significance level: *p ≤ 0.05, **p < 0.01, ***p < 0.001.

**Fig 3:**
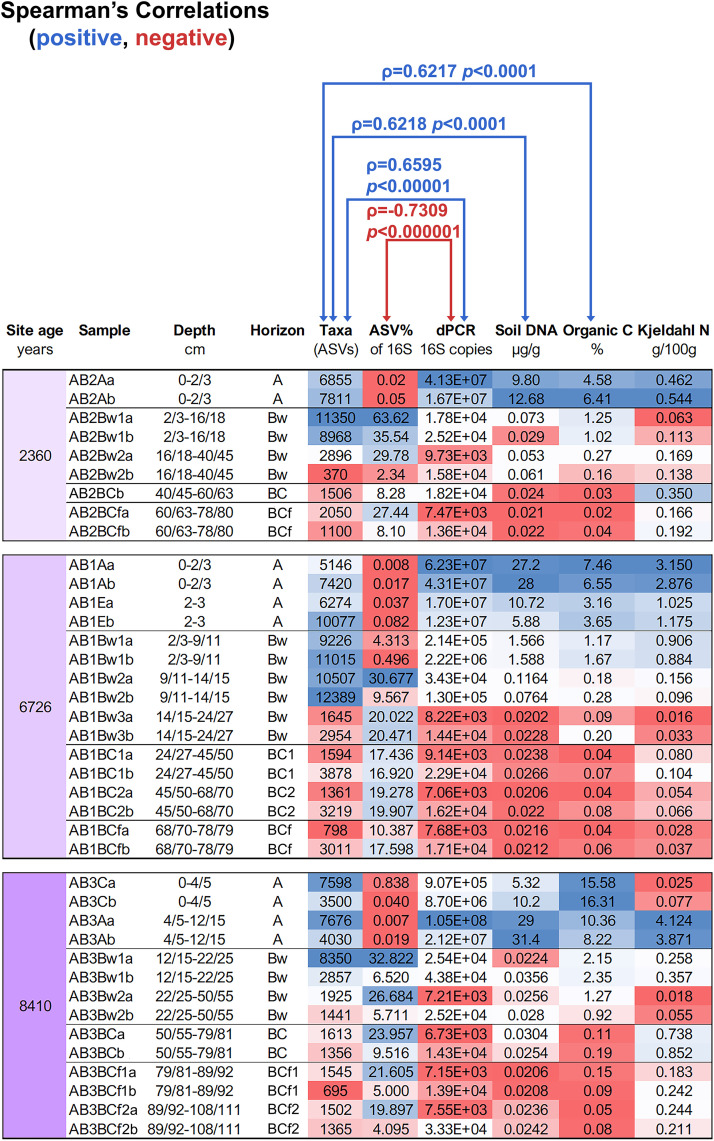
Gradients of bacterial diversity in relation to the soil biological and chemical parameters through the depth profiles in the three raised beaches. ASV: Amplicon Sequence Variants (function of the taxa diversity); dPCR 16S copies: estimate of the number of bacterial ribosomal operon gene copies obtained by the digital PCR quantitation from the total DNA extracted from soil. Organic C. and Kjeldahl N: organic carbon percentage and total nitrogen, respectively, measured in the soil samples. The DNA extraction was performed on a single bulk sample core for the two replicates. The non-parametric Spearman’s Rank correlation coefficient (Spearman’s ρ) was calculated from individual pairs of data columns and some of the significant outcomes are indicated along with the associated p values. The Microsoft Excel conditional formatting (blue to red shading) is applied to the data matrix to better visualize the differences.

To further examine differences in microbial diversity and the community divergence occurring throughout the profile of each site, detailed data are shown in Fig 3. The number of different sequence variants reached its maximum in the layer between 2/3 and the 15/20 cm of depth and to undergo an abrupt step of decrease localized at the transition below that distance from the surface. Moreover, under that level, the number of ASVs is steadily kept throughout the horizons, including those in the permafrost. This layer (corresponding to the upper Bw horizon of the two youngest beaches) does not coincide with the highest OC or soil DNA content, which tend to be localized at the top of each site. Considering the whole profile, a positive correlation between the number of ASVs and either soil carbon, soil DNA, or 16S gene copies is detected (blue arrows in Fig 3) but, interestingly, a negative correlation between ASVs and their own percentage of the 16S gene copies is also displayed. This pattern suggests that the niches where the detectable bacterial diversity is higher are those where bacterial abundance is lower. This allows us to suppose that when microbial communities contain many individuals (i.e., supposedly they result from active processes of growth), the abundance of many having the same taxonomic identity can obscure the possibility of detecting the rare ones.

Concerning the values of TKN, no significant correlation for *p* < 0.05 resulted with ASVs number (ρ = 0.30, p value = 0.069). Moreover, as shown in Fig 3, calculations indicate that, in most horizons, the ratio between OC and total N resulted either much higher or much lower than the ratio 10/1 value that typical soils hosting processes of microbial resynthesis of plant litter would display in temperate regions.

Correlation analysis among biological and physicochemical parameters (S1 Fig.) revealed strong relationships. The number of 16S gene copies showed robust correlations with all soil parameters. In detail, it was positively correlated with OC (ρ = 0.78; *p* < 0.001), available P (ρ = 0.72; *p* < 0.001), and total soil DNA (ρ = 0.84; *p* < 0.001), and negatively correlated with soil pH (ρ = −0.76; *p* < 0.001).

**Fig 4:**
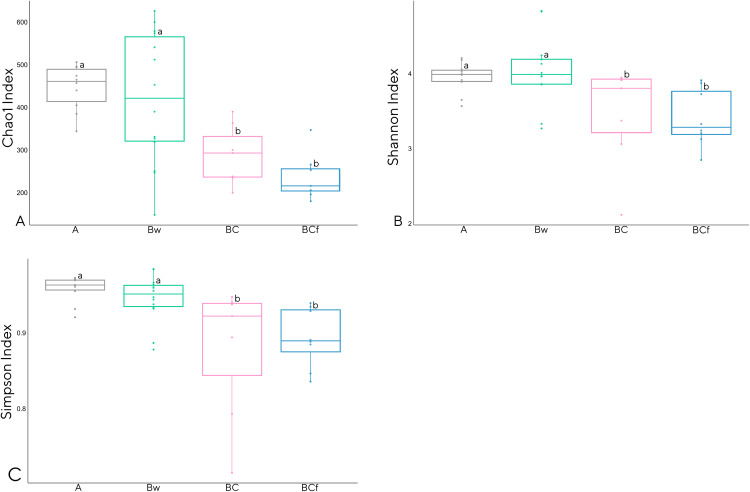
Boxplot comparison of three alpha-diversity ecological indices calculated using the genus taxonomic level in the identified horizons. A horizon in grey, Bw horizon in green, BC horizon in pink, and F horizon in blue. **(A)** Taxa richness calculated using the Chao1 index, **(B)** Shannon diversity index, **(C)** Simpson diversity index.

**Fig 5:**
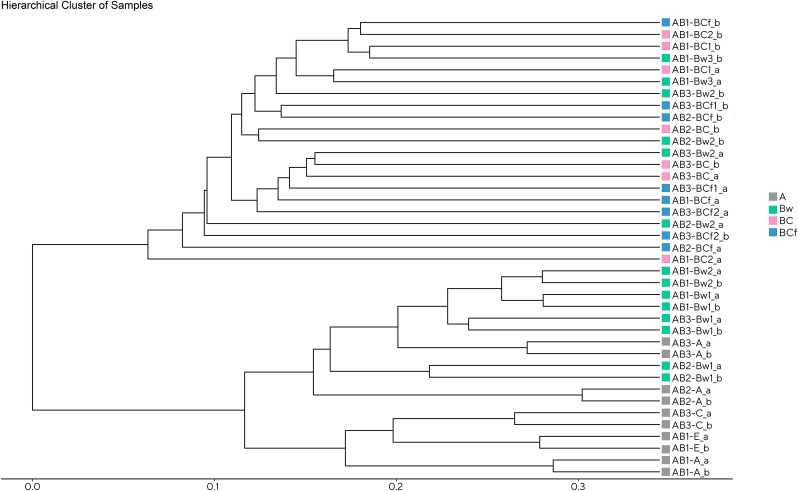
Hierarchical cluster dendrogram of samples based on the Bray-Curtis dissimilarity matrix. Soil samples displayed in grey belong to the A horizon, samples displayed in green belong to the Bw horizon, samples displayed in pink belong to the BC horizon, and samples displayed in blue belong to the BCf horizon.

Regarding taxonomic composition, the 106,175 identified ASVs were classified into 1,425 taxonomically annotated names. At the phylum rank level, the classification covered 76% of the annotated reads, while at the class level it encompassed 75%. Further, at the order level, the classification covered 73% of the reads and, at the family and genus levels, it reached 72% and 68%, respectively. Metabarcoding data analyses results are presented at the genus rank level, which provides a representative overview of higher taxonomic ranks. Alpha diversity within every taxonomical level, from phylum to genus, was assessed through the computation of three ecological indices, specifically Chao1, Shannon, and Simpson 1-D. These indices were employed to evaluate community richness and diversity. Site-based clustering (S2 Fig.), although not pointing out a significance of the observed differences, showed that the AB1 soil exhibited the highest absolute value of genera richness, while AB2 showed the broadest range of values. Conversely, for the Shannon and Simpson 1-D indices, the AB2 soil showed the highest absolute diversity, and AB3 soil displayed the broadest range. The horizon clustering (Fig 4) showed that, for all the considered indices, A and Bw horizons have significantly higher richness (*p* < 0.001, H = 16.89) and diversity (Shannon index: H = 14.19 and p < 0.01, Simpson index: H = 15.58 and p < 0.01) values when compared to deeper BC and BCf horizons. This clustering contraposition of superficial *vs*. deep horizons was also observed in the hierarchical cluster plot based on the Bray-Curtis dissimilarity matrix (Fig 5). To add detail on this aspect, all the pairwise intercommunity distances among samples are shown in S1 Dataset spreadsheet where the Whittaker distance matrix and the Bray-Curtis similarity matrix are shown. The mid portion of the Bw horizon sets the cutoff not only for richness, as seen in Fig 3, but also for community similarity. Moreover, the mid-aged beach, aa hotspot of identifiable community similarity (blue-shaded areas) was observed in the upper Bw horizons, corresponding to the layer that also supported the highest variety of detectable ASVs, suggesting that these areas promote taxonomic overlap and diversity.

**Fig 6:**
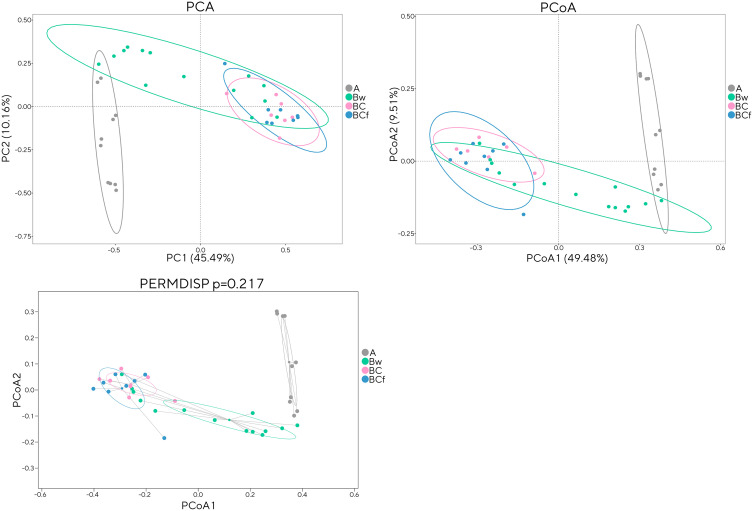
Multivariate analyses for the bacterial communities sequencing data at the genus taxonomic level to evaluate the beta diversity in the identified horizons. A horizon in grey, Bw horizon in green, BC horizon in pink, BCf horizon in blue. **(A)** Principal Component Analysis (PCA), **(B)** Principal Coordinate Analysis (PCoA) based on the Bray-Curtis dissimilarity matrix, **(C)** Permutational Multivariate Analysis of Dispersion (PERMDISP) showing the distance of each sample from the group’s centroid.

**Fig 7:**
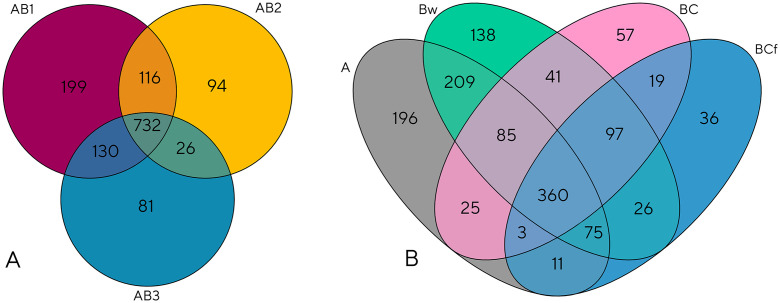
Venn diagrams reporting the shared and unique identified numbers of genera. **(A)** Core microbiome evaluated among the three raised beaches (AB1 in red, AB2 in yellow, and AB3 in blue), **(B)** core microbiome evaluated among the four horizons (A horizon in grey, Bw horizon in green, BC horizon in pink, and BCf horizon in blue).

A further beta diversity analysis was conducted to gain deeper insight into the distinctions among the studied samples and to discern their mutual relationships. The analysis again showed that the site-based clustering showed no significant differences among the three raised beaches (S3 Fig.), yielding a Permutational Multivariate Analysis of Variance (PERMANOVA) with *p*-value of 0.648 for 999 permutations, and a Permutational Multivariate Analysis of Dispersion (PERMIDSP) *p*-value of 0.288 (999 permutations). In contrast, the horizon-based clustering (Fig 6)showed a significant PERMANOVA (*p* ≤ 0.001) but a non-significant PERMDISP result. The analysis of shared and unique taxa (Fig 7) showed that, despite the homogeneity of the alpha and beta diversities among the investigated sites, each raised beach held a detectable number of unique genera. The horizon-based clustering, as well, revealed a substantial presence of unique genera characterizing each horizon. Once again, it was evident that the A and Bw horizons were distinctly differentiated from the deepest horizons in terms of abundance of unique genera and microbial community profile (S4 Fig). It should be noted that these differences, although notable, are associated with taxa that represent less than 1% of the total identified taxonomic composition. At the phylum level, the core microbiome was dominated by Actinobacteria, Proteobacteria, and Firmicutes (S5 Fig.), collectively accounting for 42%, 22%, and 18% of the total identified taxa, respectively.

The FAPROTAX online taxonomy-based predictor identified a total of 57 functions. In general, the site-based clustering did not reveal significant differences among the three raised beaches, whereas the horizon-based clustering showed a greater number of identified functions in the superficial horizons (A and Bw). These horizons also showed a lower evenness in the ASVs distribution across function (S6 Fig.).

## Discussion

### Soil formation processes

The soils at the three raised beaches originated from glacial till made of large blocks, stones, and pebbles of different nature where crystalline and dolomitic rock fragments prevailed (S2 Table). The chaotic arrangement of gravel produces soils with excessive drainage full of voids, where accumulation of fine earth occurs on the upper surface of the rock fragments when fine particles are translocated by water fluxes to form silt caps (e.g., [4,55]). It is worth noting that, because of the sandy texture of the fine earth, these accumulations should be more properly named “sand caps”, even though their morphology resembled that of silt-made accumulations; formation of sand caps by illuviation has been already reported by Agnelli et al. [56] for the soils under *gelic* temperature regime of the summit of Etna Volcano. The fine earth in the raised beach soils likely originated *in situ*, by comminution due to thermal excursion (e.g., [57]) and stone grinding (e.g., [58]), and by deposition of wind-blown materials (e.g., [59,60]). As a matter of fact, windblown dust that accumulated within stony areas and filtered through the rock fragments was documented in the landscape of Devon Island [4]. Aerial dispersion on materials includes bacteria (e.g., [61,62]) and invertebrates like mites, springtails, aphids, and other non-flying organisms for which wind transport represents a system to colonize new habitats (e.g., [63]). In correspondence of water fluxes (rain events or snow-thawing), fines can be translocated toward depth and accumulated on sub-horizontal upper surfaces of rock fragments, forming silt caps. Therefore, in these soils, the fine earth found even at depth can be the results of *in situ* and *ex situ* pedogenesis. Notwithstanding the small annual precipitation (<200 mm) and the short period of active layer thawing (<2 months, judging from the ice-melting period of the close ice cap [64]), the presence of carbonates pendants and reddish-brown staining (S1 Table) indicated the occurrence of water fluxes able not only to translocate fines, but also to solubilize and redistribute both carbonates and elements like iron.

### DNA preservation and methodological implications

Fine earth samples from horizons formed across thousands of years have yielded workable material under the molecular profiling standpoint. The substantial detectability of 16S rDNA can be attributed also to the specific environmental traits of this Arctic lowland. Devon Island undergoes minimal environmental change, has received negligible human impact, and features low precipitation levels. These factors, coupled with the propensity of certain bacteria to sporulate, collectively create favorable conditions for DNA preservation. It is important to emphasize that DNA preservation and amplifiability, while crucial for the analysis process, are not sufficient on their own to ensure reliable results. The decision to employ dPCR, a robust molecular technique known for its high biological sensitivity and reproducibility [65], enables the detection of challenging DNA quantities without the need for an external calibration curve [66,67].

### Pedoclimatic conditions and aeolian contribution to microbial patterns

The assessed MN enrichment in AB3 beach soil is peculiar and mostly ascribable to the presence of micas; however, probably driven by the non-deterministic variation in the mineral composition of the beach itself, the absence of significant differences among the investigated sites can be attributed to their common location within the Devon Island lowland, which exert consistent ecological pressures on soil microbial communities. The quantifiable observed differences regarding unique taxa and predicted functions can be attributed to a passive enrichment in microbial biomass driven by wind action [68], and such aeolian transportation is an utterly relevant phenomenon well-documented in the Canadian Arctic region [69–72], and it has also been recorded in the Devon Plateau [4].

### Abiotic drivers of microbial community structure

Extensive research has provided substantial evidence regarding the pivotal role played by abiotic factors in shaping the composition and structure of soil bacterial communities [73,74,75,76,77]. In line with the existing literature, our results highlight that physicochemical differences among horizons actively shape microbial community composition and structure. In particular, the hierarchical cluster analysis based on the Bray-Curtis dissimilarity matrix (Fig. 5) vividly illustrates the distinct separation between surface and deep horizons. The PCA biplot of Fig. 2 and the corrplot of S1 Fig. point out at pH, OC content, and available P content as associated to the soil microbiome. pH is acknowledged as a key regulator of nutrient availability in soil, thus emerging as the primary influencer of bacterial community diversity, even within Arctic soils [78,79]. We observed a robust negative correlation between soil pH and the levels of OC and available P. Simultaneously, it reveals a strong positive correlation linking total soil DNA content, 16S gene copies, and the contents of OC and available P, in accordance with the results published by Tian et al. [80] and Oliviero et al. [81]. The observed correlations are indeed consistent with the lower pH featured by OC and with the fact that organic matter is structurally coincident with P, DNA, and 16S-bearing bacteria. The analysis of beta diversity, specifically employing PCA and PCoA coupled with PERMANOVA, revealed that the observed diversities in the average community composition within each horizon are statistically significant. The identification of a core microbiome comprising Actinobacteria, Proteobacteria, and Firmicutes aligns with prior research conducted in Arctic [82] and Antarctic [83,84] polar regions. These phyla, which appear to play crucial roles in the circulation of C, N, P, and sulphur [85–88], are not confined to polar regions alone. They can also be found in highly disturbed environments, including hot deserts [89,90], grazed soils [91], and soils impacted by controlled and wild-fires [92]. More generally, however, it can be also observed that these dominant phyla are actually always among the top players in overall microbiome surveys almost everywhere [93], occurring both in temperate mesophilic as well as in extremophilic environments, and, as such, they do not entail the nature of particularly bio-indicative proxies for Arctic areas or psychrophilic habitats.

### Vertical distribution of diversity

Data presented in Fig 3 reveal a boundary within the vertical soil profiles of all three raised beaches, marking a distinct peak in microbial diversity. This boundary occurs at a depth of roughly 2–3–15–20 cm, corresponding more closely to a soil thickness than to a formal horizon, although it generally coincides with the upper part of the Bw horizon.

To better interpret this pattern, we examined the ratio between ASVs identified through metabarcoding and total 16S gene copies quantified by qPCR. The observed negative correlation between these two parameters suggests that high abundances of a few dominant taxa may mask the detection of less abundant members, leading to an underestimation of true diversity. This ratio thus provides a useful indicator for identifying communities where apparent diversity may be constrained by uneven taxonomic dominance.

Equally noteworthy, and possibly linked to this, is the stability of diversity across deeper soil layers (below 20 cm to 80 cm in the oldest site), where a decline in microbial richness would ordinarily be expected under increasingly inhospitable environmental conditions. Even the transition from the upper Bw to its underlying layers does not exhibit a marked decrease in ASVs richness. This allows us to hypothesize that the detected DNA in the deepest horizons likely reflects preserved or relic genetic material rather than signatures of active microbial metabolism. It needs to be added that, however, considering the well-known geologic, climatic, and vegetational history of these sites since their earliest emergence after deglaciation, it is plausible that both the upper and lower horizons have remained largely devoid of sustained biological activity, functioning instead as long-term repositories of dormant of relic microbial remnants.

### Unexpected microbial richness and relic DNA hypothesis

A striking feature of our dataset is the high alpha diversity observed across all samples in absolute terms, averaging 4,587 ASVs per sample. Although decreasing through the vertical profile, the community DNA still featured considerable diversity levels also in the permafrost horizon, which harbored a substantial number of taxa, exceeding 1,500 ASVs per sample. These findings raise a critical interpretative issue concerning the evidence that a massive portion of soil microbial DNA may belong to relics from dead or inactive cells rather than living organisms, and thus not related to active ecosystem processes and potentially misleading their interpretation [94]. Such relic DNA can account for up to 80% of the total microbial DNA content in soils [95]. When compared with temperate soils, including those under conventional or organic management [96], as well as soils undergoing recovery after degradation and reconstitution [37], and analysed using the same sequencing method and bioinformatics approach, we obtained rather similar levels of taxa diversity across all soil types despite the markedly different environmental conditions. An arising question in this respect is whether soil metabarcoding surveys truly reflect the level of live metabolic activity, or are instead biased by a bulk of inactive DNA accumulated over millennia through microbial discharge from atmospheric circulation and other phenomena unrelated to the actual ecosystem processes?

## Conclusions

The results presented in this work contribute to expanding our knowledge of microbial communities characterizing the High Arctic environment and their potential relationships with the local conditions. Furthermore, data reinforce existing evidence on the persistence and preservability of environmental DNA, confirming that it remains a relatively stable and detectable molecule even in temporally remote soil horizons of this kind. Additionally, these findings contribute to broadening current knowledge on biota occurring in extreme settings, and support predictions on how climate changes may influence soil microbiome, with implications for soil health and stability in remote environments. At the same time, they foster the discussion on the extent to which metabarcoding approaches truly reflect ongoing physiological activities, as opposed to representing a mere archival record of buried DNA residues accumulated over time through passive dispersion. Determining how closely 16S and ITS-derived information correspond to current ecological processes, rather than to a background of the continuous deposited airborne cells, remains a critical issue for soil studies across all latitudes.

## Supporting information

S1 Fig Spearman’s rank correlation plot reporting the correlation coefficient ρ for alle the correlations among chemical and biological parameters. Blue and red colours indicate positive and negative correlation, respectively. Significance level p ≤ 0.001.
(PNG)

S2 Fig Boxplot comparison of three alpha-diversity indices calculated at the genus taxonomic level in the three raised beaches. AB1 in red, AB2 in yellow, and AB3 in blue. (A) Taxa richness calculated using the Chao1 index, (B) Shannon index, (C) Simpson index.
(PNG)

S3 Fig Multivariate analyses for the bacterial communities sequencing data at the genus taxonomic level to evaluate the beta diversity in the three raised beaches. AB1 in red, AB2 in yellow, and AB3 in blue. (A) Principal Component Analysis (PCA), (B) Principal Coordinate Analysis (PCoA) based on the Bray-Curtis dissimilarity matrix, (C) Permutational Multivariate Analysis of Dispersion (PERMDISP) showing the distance of each sample from the group’s centroid.
(PNG)

S4 Fig Relative sequence abundance of bacterial genera associated within each sample. The top 30 most abundant genera are displayed individually, the rest of the identified genera are marked as “others”.
(PNG)

S5 Fig Relative sequence abundance of bacterial phyla associated within each sample. The top 30 most abundant phyla are displayed individually, the rest of the identified phyla are marked as “others”.
(PNG)

S6 Fig Relative abundance of the predicted functions identified within each soil sample by the FAPROTAX database. All the fifty-seven functions are displayed individually.
(PNG)

S1 Table Morphology of the soils at the raised beaches at “seagull beach” area, Devon Island Truelove Lowland, High Arctic Canada. Codes according to Schoeneberger et al. [30].
(DOCX)

S2 Table Pebble count of the soils at the raised beaches at “seagull beach”, Devon Island Truelove Lowland, High Arctic Canada. (DOCX)

S3 Table Chemical composition of each layer in the three investigated sites at “seagull beach”, Devon Island Truelove Lowland, High Arctic Canada. (DOCX)

S4 Table Skeleton and texture contents of the three investigated sites at “seagull beach”, Devon Island Truelove Lowland, High Arctic Canada. Mean and standard deviation (SD) values from two replicates are reported; values of clay were below the detection thresholds.
(DOCX)

S1 Dataset Spreadsheet where the Whittaker distance matrix and the Bray-Curtis similarity matrix are shown. (XLSX)
