# Supplementary material for: Evaluation of the soil microbiome of three raised beaches in the Devon Island Lowland, High Arctic, Canada
Source: PLoS One. 2025 Nov 5;20(11):e0336235. doi: 10.1371/journal.pone.0336235 (PMC12588476; doi:10.1371/journal.pone.0336235)
Supplement: S1 Table — Codes according to Schoeneberger et al. [30]. (DOCX) [file pone.0336235.s007.docx]

**Evaluation of the soil microbiome of three raised beaches in the Devon Island Lowland, High Arctic, Canada**

Laura Maretto, Saptarathi Deb, Andrea Squartini, Giuseppe Concheri, Piergiorgio Stevanato, Serenella Nardi, Stefania Cocco, Giuseppe Corti

Corresponding author: Laura Maretto, laura.maretto@unipd.it

| **Table S1**. Morphology of the soils at the raised beaches at “seagull beach” area, Devon Island Truelove Lowland, High Arctic Canada. Codes according to Schoeneberger et al. [30]. | | | | | | | | | | |
| --- | --- | --- | --- | --- | --- | --- | --- | --- | --- | --- |
| Horizon | Depth | Color^a^ | Structure^b^ | Consistence^c^ | Roots^d^ | Boundary^e^ | Silt caps^f^ | Carbonate pendants^g^ | Staining^h^ |  |
|  | cm |  |  |  |  |  |  |  |  |  |
| General information. Wet tundra biome interrupted by beaches raised following glacio-isostatic crustal rebound. High Arctic Canada.  Exposure: W. Mean annual air temperature: -16.0°C. Mean annual precipitation (including snow water equivalent): 185 mm. Coldest month: January (-28.5°C). Warmest month: July (5°C) [24, 26, 53]. Dating according to Kelly and King [29]. | | | | | | | | | | |
| **Beach AB2, 2360 Years Before Present**  Elevation: 3.22 m. Surface: no desert pavement; 50-60% of the surface is covered by pebbles and stones with crusty lichens, 7-10% covered by vascular plants (*Cerastium* sp., *Saxifraga* sp., *Salix* sp.), 30-40% is barren. Parent material: marine reworked glacial till. Soil: sandy-skeletal, mixed, hypergelic, Typic Haplorthel*. | | | | | | | | | | |
| A | 0-2/3 | 10YR 3/2 | v1th pl →f cr | m vfr, w ss, po | 3mi, vf, f | CI | - | 1, + | - |  |
| Bw1 | 2/3-16/18 | 10YR 4/4 | sg | w so, po | 3mi, vf, f, 1 co | CS | + | 2, +++ | 2.5YR 3/2 |  |
| Bw2 | 16/18-40/45 | 10YR 5/5 | sg | w so, po | 2mi, vf, f | CS | ++ | 3, ++++ | 2.5YR 3/4 |  |
| BC | 40/45-60/63 | 10YR 5/3 | sg | w so, po | 1mi, vf, f | CS | +++ | 1, + | - |  |
| BCf | 60/63-78/80 | 10YR 5/4 | sg | w so, po | 0 | - | + | - | - |  |
| **Beach AB1, 6726 Years Before Present**  Elevation: 22.44 m. Surface: moderately developed desert pavement; 30-40% of the surface is covered by pebbles and stones with crusty lichens, 7-10% covered by vascular plants (*Dryas* sp., *Saxifraga* sp., *Salix* sp.), 50-60% is barren. Parent material: marine reworked glacial till. Soil: sandy-skeletal, mixed, hypergelic, Typic Haplorthel*. | | | | | | | | | | |
| A | 0-2/3 | 5YR 2/2 | 1f cr | m fr, w ss, po | 3mi, vf, f | AB | - | 1, ++ | 2.5YR 4/3 |  |
| E | 2-3 | 10YR 5/1 | sg | w so, po | 3mi, vf, f | AB | - | - | - |  |
| Bw1 | 2/3-9/11 | 10YR 3/4 | 1th pl → f cr | m vfr, w so, po | 3mi, vf, f, 2m | CS | ++ | 3, ++++ | 2.5YR 3/2 |  |
| Bw2 | 9/11-14/15 | 10YR 1/2 | 1f cr | m vfr-fr, w so, po | 2mi, vf, f | CI | ++ | 2, ++++ | 2.5YR 3/2 |  |
| Bw3 | 14/15-24/27 | 10YR 4/3 | sg | w so, po | 2mi, vf, f | CW | ++++ | 4, ++++ | 2.5YR 3/3 |  |
| BC1 | 24/27-45/50 | 10YR 4/2 | sg | w so, po | 1vf, f, m | CS | +++++ | 3, ++++ | 2.5YR ¾ |  |
| BC2 | 45/50-68/70 | 10YR 5/2 | sg + m | m vfr, w so, po | 0 | CS | - | 2, + | - |  |
| BCf | 68/70-78/79 | 10YR 5/2 | sg | w so, po | 0 | - | - | - | - |  |
| **Beach AB3, 8410 Years Before Present**  Elevation: 47.52 m. Surface: well-developed desert pavement; 10-20% of the surface is covered by crusty lichens, 7-10% covered by vascular plants (*Dryas* sp., *Saxifraga* sp., *Salix* sp.), 70-80% is barren. Parent material: marine reworked glacial till. Soil: sandy-skeletal, mixed, hypergelic, Typic Haplorthel*. | | | | | | | | | | |
| C | 0-4/5 | 7.5YR 6/4 | sg | w so, po | 0 | AI | - | 1, + | 2.5YR 3/2 |  |
| A | 4/5-12/15 | 2.5YR 3/2 | 1f cr | m fr, w ss, po-sp | 3mi, vf, f, 1co | AI | - | 2, +++++ | 2.5YR 4/3 |  |
| Bw1 | 12/15-22/25 | 10YR 5/3 | sg | w so, po | 2mi, vf, f | AI | +++++ | 3, +++++ | 2.5YR 3/2 |  |
| Bw2 | 22/25-50/55 | 10YR 5/3 | sg | w so, po | v1mi, f | CS | +++++ | 4, +++++ | 2.5YR 3/3 |  |
| BC | 50/55-79/81 | 10YR 5/3 | sg | w so, po | 0 | CS | ++++ | 1, + | - |  |
| BCf1 | 79/81-89/92 | 10YR 5/2 | sg | w so, po | 0 | CS | - | - | - |  |
| BCf2 | 89/92-108/111 | 10YR 5/2 | sg | w so, po | 0 | - | - | - | - |  |

* Soil classification according to Soil Survey Staff, (2014).

^a^ Moist and crushed, according to the Munsell Soil Colour Chart (1954 edition).

^b^ v1=very weak, 1=weak; f=fine, m=medium, th=thin; cr=crumb, pl=platy; sg=single grain.

^c^ m=moist, w=wet; vfr= very friable, fr=friable; so=nonsticky, ss=slightly sticky, po=nonplastic, sp=slightly plastic.

^d^ 0=absent, v1=very few, 1=few, 2=common, 3=many; mi=micro, vf=very fine, f=fine, m=medium, co=coarse.

^e^ A=abrupt, C=clear; B=broken, W=wavy, S=smooth, I=irregular.

^f^ +=poorly developed, 1-2 mm thick; ++=poorly to moderately developed, 3-4 mm thick; +++=moderately developed, 5-6 mm thick; ++++=moderately to strongly developed, 7-8 mm thick; +++++=strongly developed, > 8 mm thick.

^g^ 1=few, interesting < 10% of the below-surface of the stones; 2=common, interesting 10-50% of the below-surface of the stones; 3=diffused, interesting 50-90% of the below-surface of the stones; 4=strongly diffused, >90% of the below-surface of the stones; +=poorly developed, 1 mm long; ++=poorly to moderately developed, 2 mm long; +++=moderately developed, 5-6 mm thick; ++++=moderately to strongly developed, 7-8 mm thick; +++++=strongly developed, > 8 mm thick.

^h^ According to the Munsell Soil Colour Chart [32].
