# Supplementary material for: Evaluation of the soil microbiome of three raised beaches in the Devon Island Lowland, High Arctic, Canada
Source: PLoS One. 2025 Nov 5;20(11):e0336235. doi: 10.1371/journal.pone.0336235 (PMC12588476; doi:10.1371/journal.pone.0336235)
Supplement: S2 Table — (DOCX) [file pone.0336235.s008.docx]

**Evaluation of the soil microbiome of three raised beaches in the Devon Island Lowland, High Arctic, Canada**

Laura Maretto, Saptarathi Deb, Andrea Squartini, Giuseppe Concheri, Piergiorgio Stevanato, Serenella Nardi, Stefania Cocco, Giuseppe Corti

Corresponding author: Laura Maretto laura.maretto@unipd.it

| **Table S2**. Pebble count of the soils at the raised beaches at “seagull beach”, Devon Island Truelove Lowland, High Arctic Canada. | | | | | |
| --- | --- | --- | --- | --- | --- |
| Horizon | Crystalline | Dolomitic | Sandstone | Diabase | Breccia + Shales |
| **AB2 beach, 2360 years before present** | | | | | |
| A | 35.5 | 56.3 | 6.2 | 0.0 | 2.0 |
| Bw1 | 23.7 | 73.4 | 1.3 | 0.0 | 1.6 |
| Bw2 | 19.7 | 76.9 | 2.9 | 0.0 | 0.5 |
| BC | 21.0 | 70.3 | 7.7 | 0.0 | 1.0 |
| BCf | 21.8 | 69.0 | 5.5 | 0.0 | 3.7 |
| **AB1 beach, 6726 years before present** | | | | | |
| A | 47.0 | 7.4 | 20.5 | 2.1 | 23.0 |
| E | 53.6 | 10.2 | 9.6 | 0.0 | 26.6 |
| Bw1 | 54.5 | 39.3 | 4.7 | 0.0 | 1.5 |
| Bw2 | 51.0 | 36.6 | 2.4 | 0.6 | 9.4 |
| Bw3 | 46.5 | 32.0 | 1.0 | 1.7 | 18.8 |
| BC1 | 51.2 | 41.5 | 1.3 | 2.4 | 3.6 |
| BC2 | 47.0 | 41.4 | 0.5 | 0.8 | 10.3 |
| BCf | 42.9 | 38.0 | 1.0 | 1.2 | 16.9 |
| **AB3 beach, 8410 years before present** | | | | | |
| C | 84.9 | 12.5 | 1.0 | 0.0 | 1.6 |
| A | 61.9 | 17.5 | 18.6 | 0.0 | 2.0 |
| Bw1 | 68.3 | 26.4 | 3.3 | 0.0 | 2.0 |
| Bw2 | 68.0 | 29.0 | 0.0 | 0.0 | 3.0 |
| BC | 61.8 | 22.1 | 9.1 | 5.1 | 1.9 |
| BCf1 | 61.6 | 31.6 | 1.1 | 5.0 | 0.7 |
| BCf2 | 59.5 | 31.0 | 3.9 | 3.8 | 1.8 |
