# Supplementary material for: Evaluation of the soil microbiome of three raised beaches in the Devon Island Lowland, High Arctic, Canada
Source: PLoS One. 2025 Nov 5;20(11):e0336235. doi: 10.1371/journal.pone.0336235 (PMC12588476; doi:10.1371/journal.pone.0336235)
Supplement: S3 Table — (DOCX) [file pone.0336235.s009.docx]

**Evaluation of the soil microbiome of three raised beaches in the Devon Island Lowland, High Arctic, Canada**

Laura Maretto, Saptarathi Deb, Andrea Squartini, Giuseppe Concheri, Piergiorgio Stevanato, Serenella Nardi, Stefania Cocco, Giuseppe Corti

Corresponding author: Laura Maretto laura.maretto@unipd.it

| **Table S3**. Skeleton and texture contents of the three investigated sites at “seagull beach”, Devon Island Truelove Lowland, High Arctic Canada. Mean and standard deviation (SD) values from two replicates are reported; values of clay were below the detection thresholds. | | | | | | | | | | | | | | | | | | | |
| --- | --- | --- | --- | --- | --- | --- | --- | --- | --- | --- | --- | --- | --- | --- | --- | --- | --- | --- | --- |
| Horizon | Bulk density of fine earth | | Bulk density of skeleton | | Skeleton content | | Skeleton content | | Coarse sand | | Medium sand | | Fine sand | | Total sand  (2-0.05 mm) | | Silt | | Clay |
|  | g cm^-3^ | | g cm^-3^ | | by volume % | | by weight % | | (2-0.5 mm) | | (0.5-0.25 mm) | | (0.25-0.05 mm) | |  |  | (0.05-0.002 mm) | | (<0.002 mm) |
|  | Mean | SD | Mean | SD | Mean | SD | Mean | SD | Mean | SD | Mean | SD | Mean | SD | Mean | SD | Mean | SD |  |
| **Beach AB2, 2360 Years Before Present** | | | | | | | | | | | | | | | | | | | |
| A | 1.3 | 0.12 | 2.55 | 0.05 | 65.8 | 4.1 | 78.9 | 4.4 | 60.4 | 1.9 | 7.5 | 1.77 | 27.3 | 3.04 | 95.2 | 0.64 | 4.75 | 0.64 | <0.1 |
| Bw1 | 1.7 | 0.08 | 2.74 | 0.08 | 79.6 | 2.7 | 86.2 | 3.0 | 63.7 | 1.6 | 5.9 | 1.48 | 21.3 | 1.84 | 90.9 | 1.7 | 9.1 | 1.7 | <0.1 |
| Bw2 | 1.22 | 0.03 | 2.62 | 0.06 | 62.8 | 3.5 | 78.1 | 3.7 | 62.6 | 4.2 | 7.9 | 0.78 | 19.7 | 3.04 | 90.2 | 0.42 | 9.8 | 0.42 | <0.1 |
| BC | 1.32 | 0.08 | 2.66 | 0.02 | 65.1 | 4.7 | 78.1 | 3.3 | 65.9 | 4.2 | 9.6 | 1.84 | 15.2 | 1.27 | 90.7 | 1.13 | 9.3 | 1.13 | <0.1 |
| BCf | 0.81 | 0.07 | 2.66 | 0.07 | 63.9 | 4.5 | 83.6 | 6.2 | 93.8 | 2.4 | 2.9 | 0.85 | 1.1 | 0.49 | 97.8 | 1.06 | 2.25 | 1.06 | <0.1 |
| **Beach AB1, 6726 Years Before Present** | | | | | | | | | | | | | | | | | | | |
| A | 1.76 | 0.29 | 2.62 | 0.09 | 41.8 | 8.6 | 51.6 | 7.2 | 80.5 | 4.0 | 7.0 | 1.91 | 11.3 | 1.7 | 98.8 | 0.42 | 1.2 | 0.42 | <0.1 |
| E | 1.6 | 0.05 | 2.63 | 0.06 | 39.3 | 5.8 | 45.4 | 4.5 | 79.8 | 0.4 | 6.0 | 0.78 | 11.4 | 0.71 | 97.2 | 0.49 | 2.75 | 0.49 | <0.1 |
| Bw1 | 1.88 | 0.14 | 2.54 | 0.07 | 34.9 | 2.0 | 40.7 | 1.5 | 76.2 | 4.7 | 6.3 | 0.92 | 10.9 | 0.78 | 93.4 | 3.04 | 6.55 | 3.04 | <0.1 |
| Bw2 | 1.69 | 0.08 | 2.77 | 0.14 | 56.1 | 4.0 | 68.0 | 4.0 | 78.8 | 3.5 | 7.3 | 0.57 | 10.8 | 2.05 | 96.9 | 0.85 | 3.1 | 0.85 | <0.1 |
| Bw3 | 1.74 | 0.05 | 2.72 | 0.09 | 66.5 | 3.0 | 81.2 | 5.4 | 81.2 | 0.5 | 7.2 | 0.35 | 9.6 | 1.06 | 98.0 | 0.21 | 1.95 | 0.21 | <0.1 |
| BC1 | 1.95 | 0.08 | 2.65 | 0.05 | 63.3 | 4.9 | 70.3 | 6.2 | 84.7 | 1.6 | 7.4 | 1.13 | 6.0 | 0.85 | 98.1 | 0.35 | 1.85 | 0.35 | <0.1 |
| BC2 | 2.16 | 0.08 | 2.52 | 0.09 | 75.4 | 5.6 | 78.1 | 4.9 | 84.3 | 0.8 | 8.2 | 0.71 | 5.8 | 0.85 | 98.3 | 0.71 | 1.7 | 0.71 | <0.1 |
| BCf | 0.92 | 0.06 | 2.59 | 0.12 | 85.4 | 4.4 | 91.6 | 4.5 | 92.7 | 0.7 | 3.3 | 0.78 | 2.3 | 0.57 | 98.3 | 0.64 | 1.65 | 0.64 | <0.1 |
| **Beach AB3, 8410 Years Before Present** | | | | | | | | | | | | | | | | | | | |
| C | - | - | 2.43 | 0.07 | 92.4 | 5.5 | 95.4 | 1.1 | 84.3 | 1.1 | 9.7 | 0.71 | 6.0 | 1.77 | 100 | 0 | <0.1 | <0.1 | <0.1 |
| A | 1.14 | 0.07 | 2.36 | 0.18 | 42.1 | 4.0 | 61.1 | 5.9 | 70.2 | 4.9 | 10.0 | 0.99 | 11.3 | 2.97 | 91.5 | 0.92 | 8.55 | 0.92 | <0.1 |
| Bw1 | 1.68 | 0.15 | 2.49 | 0.2 | 57.6 | 4.4 | 70.0 | 3.5 | 76.4 | 6.5 | 7.8 | 1.63 | 7.7 | 1.41 | 91.9 | 6.72 | 8.05 | 6.72 | <0.1 |
| Bw2 | 1.78 | 0.08 | 2.52 | 0.17 | 55.9 | 3.6 | 60.3 | 3.0 | 84.1 | 0.5 | 9.6 | 1.13 | 4.1 | 1.41 | 97.8 | 0.21 | 2.25 | 0.21 | <0.1 |
| BC | 1.8 | 0.08 | 2.6 | 0.06 | 45.7 | 5.2 | 55.7 | 6.1 | 89.4 | 1.9 | 5.8 | 1.13 | 3.0 | 0.35 | 98.2 | 0.42 | 1.8 | 0.42 | <0.1 |
| BCf1 | 0.8 | 0.03 | 2.66 | 0.04 | 59.5 | 5.7 | 70.4 | 7.1 | 80.1 | 2.6 | 10.9 | 0.71 | 5.7 | 1.41 | 96.7 | 0.49 | 3.35 | 0.49 | <0.1 |
| BCf2 | 0.81 | 0.04 | 2.54 | 0.18 | 73.9 | 5.5 | 91.2 | 3.3 | 89.1 | 0.4 | 3.8 | 0.85 | 4.3 | 0.71 | 97.2 | 0.28 | 2.8 | 0.28 | <0.1 |

Mean and standard deviation (SD) values from two replicates are reported.
