# Supplementary material for: Evaluation of the soil microbiome of three raised beaches in the Devon Island Lowland, High Arctic, Canada
Source: PLoS One. 2025 Nov 5;20(11):e0336235. doi: 10.1371/journal.pone.0336235 (PMC12588476; doi:10.1371/journal.pone.0336235)
Supplement: S4 Table — Mean and standard deviation (SD) values from two replicates are reported; values of clay were below the detection thresholds. (DOCX) [file pone.0336235.s010.docx]

**Evaluation of the soil microbiome of three raised beaches in the Devon Island Lowland, High Arctic, Canada**

Laura Maretto, Saptarathi Deb, Andrea Squartini, Giuseppe Concheri, Piergiorgio Stevanato, Serenella Nardi, Stefania Cocco, Giuseppe Corti

Corresponding author: Laura Maretto laura.maretto@unipd.it

| **Table S4**. Chemical composition of each layer in the three investigated sites at “seagull beach”, Devon Island Truelove Lowland, High Arctic Canada. | | | | | | | | | | |
| --- | --- | --- | --- | --- | --- | --- | --- | --- | --- | --- |
| Horizon | pH_(H2O)_ | | Organic C | | Kjeldahl N | | Micaceous N | | Available P | |
|  |  | | % | | g kg^-1^ | | mg kg^-1^ | | mg kg^-1^ | |
|  | Mean | SD | Mean | SD | Mean | SD | Mean | SD | Mean | SD |
| **Beach AB2, 2360 Years Before Present** | | | | | | | | | | |
| A | 7.57 | 0.08 | 5.50 | 1.29 | 0.50 | 0.06 | 6.0 | 0.0 | 4.0 | 1.4 |
| Bw1 | 8.02 | 0.08 | 1.14 | 0.16 | 0.09 | 0.04 | 4.5 | 0.7 | 3.0 | 0.0 |
| Bw2 | 8.15 | 0.06 | 0.22 | 0.08 | 0.15 | 0.02 | 4.5 | 0.7 | 2.5 | 0.7 |
| BC | 8.16 | 0.11 | 0.04 | 0.01 | 0.37 | 0.03 | 3.0 | 0.0 | 2.0 | 0.0 |
| BCf | 8.20 | 0.08 | 0.03 | 0.01 | 0.18 | 0.02 | 2.5 | 0.7 | 1.0 | 0.0 |
| **Beach AB1, 6726 Years Before Present** | | | | | | | | | | |
| A | 7.75 | 0.08 | 7.01 | 0.64 | 3.01 | 0.19 | 6.0 | 1.4 | 7.5 | 0.7 |
| E | 7.64 | 0.05 | 3.41 | 0.35 | 1.10 | 0.11 | 7.5 | 0.7 | 8.0 | 0.0 |
| Bw1 | 7.82 | 0.04 | 1.42 | 0.35 | 0.90 | 0.02 | 7.0 | 1.4 | 6.5 | 0.7 |
| Bw2 | 8.16 | 0.06 | 0.23 | 0.07 | 0.13 | 0.04 | 4.0 | 1.4 | 4.5 | 0.7 |
| Bw3 | 8.26 | 0.06 | 0.15 | 0.08 | 0.02 | 0.01 | 7.5 | 0.7 | 4.5 | 0.7 |
| BC1 | 8.35 | 0.02 | 0.06 | 0.02 | 0.09 | 0.02 | 6.5 | 0.7 | 3.0 | 1.4 |
| BC2 | 8.34 | 0.04 | 0.06 | 0.03 | 0.06 | 0.01 | 0.5 | 0.7 | 1.5 | 0.7 |
| BCf | 8.33 | 0.03 | 0.05 | 0.01 | 0.03 | 0.01 | 0.5 | 0.7 | 0.0 | 0.0 |
| **Beach AB3, 8410 Years Before Present** | | | | | | | | | | |
| C | 7.29 | 0.06 | 15.95 | 0.52 | 0.05 | 0.04 | 14.0 | 1.4 | 9.5 | 0.7 |
| A | 7.82 | 0.06 | 9.29 | 1.51 | 4.00 | 0.18 | 10.5 | 0.7 | 6.5 | 0.7 |
| Bw1 | 8.10 | 0.21 | 2.25 | 0.14 | 0.31 | 0.07 | 9.5 | 0.7 | 4.5 | 0.7 |
| Bw2 | 8.17 | 0.11 | 1.10 | 0.25 | 0.04 | 0.03 | 8.5 | 0.7 | 3.0 | 0.0 |
| BC | 8.21 | 0.10 | 0.15 | 0.06 | 0.80 | 0.08 | 8.5 | 0.7 | 1.5 | 0.7 |
| BCf1 | 8.32 | 0.10 | 0.12 | 0.04 | 0.21 | 0.04 | 8.5 | 0.7 | 0.5 | 0.7 |
| BCf2 | 8.54 | 0.09 | 0.07 | 0.02 | 0.23 | 0.02 | 6.0 | 1.4 | 0.0 | 0.0 |

Mean and standard deviation (SD) values from two replicates are reported.
